# Introduction to Antimicrobial Resistance and the Maternal Sepsis Intervention

**DOI:** 10.1007/978-3-030-62662-4_1

**Published:** 2020-11-24

**Authors:** Louise Ackers, Gavin Ackers-Johnson, Joanne Welsh, Daniel Kibombo, Samuel Opio

**Affiliations:** 6 Global Social Justice, University of Salford, Salford, UK; 7 University of Salford, Salford, UK; 8 Infectious Disease Institute, Kampala, Uganda; 9 Pharmaceutical Society of Uganda, Kampala, Uganda

**Keywords:** Antimicrobial resistance, Antimicrobial stewardship, Infection prevention control, Universal health coverage, International development, Global health

## Abstract

This chapter describes the threat to global health and security caused by the growing resistance of infectious organisms to antibiotics or antimicrobial resistance (AMR). Growing global connectivity ensures that AMR is a threat to us all wherever we are and with specific impacts on Low- and Middle-Income Countries (LMICs). The chapter outlines international responses to AMR including the Global Action Plan and the impact this has had on one LMIC; Uganda. It then introduces a recent UK funding call focused on improving the management of antibiotics or ‘Antimicrobial Stewardship’.

## Antimicrobial Resistance; A Post-antibiotic Apocalypse?^1^

Infection of the human body by an external vector, often a microorganism, has been a major cause of human illness and death. The development of antibiotics and their therapeutic use since the 1940s has played a critical role in the prevention and treatment of infectious disease. So much so, that there is a growing call for a shift in emphasis in global health, towards investment in non-communicable diseases such as heart disease, cancer or diabetes. Evidence of growing resistance of infectious microorganisms to commonly used antibiotics has raised serious concerns about the ability to continue to control infection and manage communicable disease. Microorganisms, including bacteria, can constantly mutate and resist the presence of antibiotics. This mutation is a naturally occurring process, but it is accelerated by the inappropriate use of antibiotics. Some of the routinely used antibiotics now have limited if any impact on common infections. Put simply, they no longer work. This has led to the development of newer antibiotics which are often more expensive for users and health systems, but many of these are now also compromised. The use of antibiotics by humans is a key factor shaping resistance; however, there is growing recognition of the impacts that the use of antibiotics in animal health—and recently in agriculture—has on human health (and vice versa). This concern is captured by the concept of ‘ One Health’, which takes a holistic view of disease and health care across both animal and human sectors.

The declining effectiveness of antibiotics in the treatment of infection not only affects communicable disease; it also impacts our ability to treat conditions such as cancer, as invasive treatment using chemotherapy, for example, generates low levels of immunity leaving patients vulnerable to infection.

The World Health Organization (WHO) defines antimicrobial resistance (or AMR) as the process of resistance that occurs when microorganisms (such as bacteria, fungi, viruses, and parasites) change when they are exposed to antimicrobial drugs (such as antibiotics, antifungals, antivirals, antimalarials, and anthelmintics). Microorganisms that develop antimicrobial resistance are sometimes referred to as ‘superbugs’. As a result of growing resistance, the medicines become ineffective and infections persist in the body, increasing the risk of spread to others.^2^

A UK Review of antimicrobial resistance chaired by Jim O’Neill describes AMR as a ‘Terrible Scenario’ posing a genuine economic and security threat (2016: 1). With the potential to reverse decades of medical progress in the treatment of communicable disease, AMR is one of the biggest threats to global health, food security and development in the world today. O’Neill reported that currently there are 700,000 global deaths a year related to AMR which is estimated to reach as high as 10 million by 2050 (O’Neill 2016). Whilst the numbers quoted were met with some scepticism due to the patchiness of data available, they give major cause for concern. Whilst human health is a priority, AMR will also have an inevitable impact on the global economy. The same study estimated that between 2015 and 2050 AMR could cost globally US$100 trillion and a study published by the World Bank estimated a reduction in global GDP of between 1.1 and 3.8 percentage points between 2017 and 2050 due to AMR (World Bank 2017).

AMR is a complex phenomenon. Science communication has failed to increase public awareness of AMR, its causes, and the need for behaviour change. A popular misconception amongst those people who have a limited understanding is that it is the individual consuming the antibiotics that become immune to their effects, rather than the microorganisms. This can lead to a level of complacency that we have the power, through our own actions, to protect ourselves from AMR by reducing our own use of antibiotics and saving them for when we really need them. Whilst this can lead to under-consumption and put some individuals at risk, it also discourages a more global, collective, consciousness.

A report on antibiotic resistance by the Ugandan National Academy of Sciences (2015) refers immediately to the impact of ‘increasing global connectivity’ resulting not only in the movement of people, animals and trade, but also ‘the rapid transport of infectious agents and their antibiotic resistant genes’ (2015: iii). Antibiotic-resistant genes do not respect international borders and spill-over to compromise global health. O’Neill’s report identified several ‘market failures’ affecting AMR. These included externality (or spill-over) effects where action or lack of action in one location impacts others. The authors argue that the rise of AMR is a global problem that cannot be addressed materially without a critical mass of countries coming together to implement consistent solutions;

Drug resistant infections spread very quickly; a person carrying a drug-resistant bacterium can fly across the world in a matter of hours. Even if out of pure self-interest, it may make sense for high-income countries to support these efforts in lower income settings. (2016: 65)

Grounded in understandable self-interest, high-income countries are increasingly aware and concerned about growing evidence of the relationship between global health threats and political and economic instability (Saha and Alleyne 2018). The experience of the global COVID-19 pandemic has triggered an overwhelming awareness of global interconnectivity. Human behaviour and antibiotic consumption practices (amongst other things) in low- and middle-income countries (LMICs) immediately impact public health in high-income countries (HICs). The converse is of far greater concern as the over-consumption of antibiotics in more affluent countries by humans, animals and plants leads to a reliance upon newer, more expensive antibiotics beyond the reach of many people in LMICs, dependent on impoverished public services.

There is also a more normative concern, articulated by international organisations such as the United Nations and the World Health Organization, and underpinned by ethical commitments to concepts of regional equality and individual equity. The Sustainable Development Goals (SDGs)^3^ were launched by the United Nations in 2016. SDG3 captures the commitment to ensuring healthy lives and well-being for all. Although SDG3 refers specifically to the need to combat epidemics and communicable diseases, there is no specific reference to the impact of AMR. Objective 3.8 enshrines a growing commitment, in global health, to ‘ universal health coverage, including financial risk protection, access to quality essential health-care services and access to safe, effective, quality and affordable essential medicines and vaccines for all’. AMR presents a major, if silent, threat to the achievement of universal health coverage in LMICs.

## Addressing Antimicrobial Resistance on a Global Platform

Alongside surveillance of resistance patterns, there is an emerging consensus that efforts to contain the threat of AMR should focus on four key objectives. The first is to address the inappropriate use of antibiotics in both human and animal populations, with high usage being the predominant factor in the development of AMR. Secondly, the effective treatment of liquid waste and sewage to reduce the volumes of antibiotics being dispersed into the environment. Thirdly, through utilising effective infection prevention and control (IPC) strategies in conjunction with water, sanitation and hygiene (WASH) programmes, the need for antibiotic usage can be reduced. Finally, it is essential that effective, affordable, and regulated antibiotics are available to those that require them to avoid the self-prescription of uncertified alternatives. These objectives are most effective in combination with new diagnostic and treatment technologies (Rochford et al. 2018).

In May 2015, the World Health Assembly endorsed a Global Action Plan (GAP) to tackle antimicrobial resistance, including antibiotic resistance, the most urgent drug resistance trend.^4^ To achieve this goal, the GAP set out five Strategic Objectives:

1. Improve awareness and understanding of antimicrobial resistance.
2. Strengthen knowledge through surveillance and research.
3. Reduce the incidence of infection.
4. Optimise the use of antimicrobial agents.
5. Develop the economic case for sustainable investment that takes account of the needs of all countries, and increase investment in new medicines, diagnostic tools, vaccines, and other interventions.

The intention was that the GAP would be aligned to the national context of each member country that would then define a National Action Plan for implementation purposes.

## The Ugandan Antimicrobial Resistance National Action Plan

The Ugandan National Action Plan was published in December 2018. Echoing the Global Action Plan (GAP), it takes a ‘whole-of-society engagement’ approach, embracing the *One Health* Agenda. This ensures that AMR can be targeted at every level be it related to humans, animals or the environment. To help provide structure and enhance collaboration, a multidisciplinary National Antimicrobial Resistance Sub-Committee (NAMRSC) was formed which comprises of representatives from multiple industries including fisheries and agriculture, water and waste management, human health, wildlife, science and research and professional societies. Through the utilisation of such sub-committees, it is hoped that the NAP will have a national, regional, and local impact. Mirroring GAP, Uganda’s National Action Plan specifies five key strategic objectives (GoU 2018):

1. Awareness
2. Infection Prevention and Control
3. Stewardship
4. Surveillance
5. Research and Innovation

## The Commonwealth Partnerships for Antimicrobial Stewardship (CwPAMS) Project Call

In 2018, a consortium of UK organisations including the UK Department of Health and Social Care (DHSC), the Commonwealth Pharmacists Association (CPA) and the Tropical Health and Education Trust (THET) came together under the umbrella known as the Commonwealth Partnerships for Antimicrobial Stewardship (CwPAMS)^5^ to launch a call for applications for funding. This scheme was part of the Fleming Fund, a wider programme managed by the DHSC that aims to help LMICs tackle antimicrobial resistance. Uganda was listed as one of four target countries along with Tanzania, Ghana and Zambia.

This book traces the intervention process from initial design of an application that would qualify for funding under the CwPAMS call and the subsequent intervention that took place. Significant attention is given (in Chapter 10.1007/978-3-030-62662-4_2) to project design processes in international development and the impact of funding systems on this. This is unusual in publications which often regard the funding mechanism as ‘house-keeping’ and simply acknowledge funders. We discuss it here as it provides a powerful illustration of the tensions that arise in international development between the increasingly prescriptive demands of funders, understandings of best practice in intervention research and the ethics of global partnerships. Chapter 10.1007/978-3-030-62662-4_2 outlines the objectives of the funders and the impact these had on intervention design and research/evaluation methods. It explains the reasons for focusing the work on maternal sepsis, one of the major causes of maternal mortality and antibiotic consumption in LMICs and describes the context within which the Maternal Sepsis Intervention took place; in a Regional Referral Hospital in Western Uganda.

In a further break with tradition, Chapter 10.1007/978-3-030-62662-4_3 presents the key outcomes of the intervention outlining major improvements in maternal mortality, length of patient stays, readmission rates and hospital expenditure. These outcomes are presented at the outset to facilitate a focus on change processes which we regard as the key findings that will inform a scalable intervention model. Chapter 10.1007/978-3-030-62662-4_4 outlines a key component of change. Ultimately, the optimal way of reducing unnecessary antibiotic use is to reduce the incidence of infection and, particularly the very high infection rates (or ‘adverse events’) associated directly with medical intervention and hospitalisation. The concept of infection prevention control (IPC) embraces a myriad of mundane, housekeeping practices routinely performed by grassroots cadres that are readily overlooked in highly medicalised or pharmaceuticalised agendas. Just as housework or care has been described by feminists as a ‘Labour of Love’ (Oakley 1974); constituted by numerous small tasks constantly un-done by those we serve, so too is basic IPC. It is the neglected foundation undertaken by underpaid and disempowered staff.

Chapter 10.1007/978-3-030-62662-4_4 addresses some of the issues most commonly associated with IPC; hand hygiene, waste disposal and infrastructure. It then addresses wound management as an infection control issue. The emergence of wound management as a central focus in the Maternal Sepsis Intervention proved pivotal in shaping the pathway to antimicrobial stewardship. Chapter 10.1007/978-3-030-62662-4_5 moves on to discuss how wound management formed the catalyst for laboratory engagement and the taking of samples for testing. The laboratory results then created the ‘objective’ evidence-base that facilitated and nurtured midwifery empowerment, task-shifting and multi-disciplinary team working . Chapter 10.1007/978-3-030-62662-4_6 addresses the role that pharmacy has and could increasingly play in antimicrobial stewardship in Ugandan public hospitals. The existence of laboratory results has triggered the emergence of clinical pharmacy roles with pharmacists playing an active role in multi-disciplinary teams. Chapter 10.1007/978-3-030-62662-4_7 traces the impact of this on prescribing behaviour (and individual patient benefits) and, more widely, on procurement planning and hospital policies. Whilst celebrating the progress made and viability of the model, Chapter 10.1007/978-3-030-62662-4_7 describes the structural impact that accesses to antibiotics and IPC supplies has on the realisation of optimal change. Chapter 10.1007/978-3-030-62662-4_8 reflects on the relationship between the knowledge mobilisation processes that have contributed to behaviour change at an individual and organisational level. It critiques the traditional emphasis in international development on one-off, formal, foreign-led ‘ training’ episodes and contrasts these with the more fluid, bi-lateral, approach to learning through co-working and mentoring; approaches that sit uneasily with the accounting methods favoured by funding bodies. Chapter 10.1007/978-3-030-62662-4_9 summarises the overall impacts of the Maternal Sepsis Intervention reflecting on the processes of capturing, sustaining, and spreading best practice in antimicrobial stewardship.

## Footnotes

1 Professor Dame Sally Davies, Chief Medical Officer England (2017) https://www.antibioticresearch.org.uk/chief-medical-officer-calls-new-action-antibiotic-resistance/.

2 https://www.who.int/en/news-room/fact-sheets/detail/antimicrobial-resistance.

3 https://www.un.org/sustainabledevelopment/sustainable-development-goals/.

4 https://www.who.int/antimicrobial-resistance/global-action-plan/en/.

5 https://www.thet.org/our-work/grants/cwpams/.
